# A qualitative evaluation and conceptual framework on the use of the Birth weight and Age-at-death Boxes for Intervention and Evaluation System (BABIES) matrix for perinatal health in Uganda

**DOI:** 10.1186/s12884-023-05402-1

**Published:** 2023-02-01

**Authors:** Michelle M. Dynes, Gaea A. Daniel, Valerie Mac, Brenda Picho, Alice Asiimwe, Agnes Nalutaaya, Gregory Opio, Vincent Kamara, Frank Kaharuza, Florina Serbanescu

**Affiliations:** 1 Division of Reproductive Health, Centers for Disease Control and Prevention, Atlanta, GA USA; 2 Nell Hodgson Woodruff School of Nursing, Emory University, Atlanta, GA USA; 3 Infectious Diseases Institute, Makerere University, Kampala, Uganda; 4 Baylor College of Medicine Children’s Foundation, Kampala, Uganda; 5 Uganda Ministry of Health, Kampala, Uganda; 6 Kampala International University, Western Campus, Ishaka Bushenyi, Uganda

**Keywords:** Birth weight, Maternal health, Perinatal mortality, Neonatal health, Newborn, Stillbirths, BABIES matrix, Uganda, Surveillance systems, Saving mothers, giving life

## Abstract

**Background:**

Perinatal mortality (newborn deaths in the first week of life and stillbirths) continues to be a significant global health threat, particularly in resource-constrained settings. Low-tech, innovative solutions that close the quality-of-care gap may contribute to progress toward the Sustainable Development Goals for health by 2030. From 2012 to 2018, the Saving Mothers, Giving Life Initiative (SMGL) implemented the Birth weight and Age-at-Death Boxes for Intervention and Evaluation System (BABIES) matrix in Western Uganda. The BABIES matrix provides a simple, standardized way to track perinatal health outcomes to inform evidence-based quality improvement strategies.

**Methods:**

In November 2017, a facility-based qualitative evaluation was conducted using in-depth interviews with 29 health workers in 16 health facilities implementing BABIES in Uganda. Data were analyzed using directed content analysis across five domains: 1) perceived ease of use, 2) how the matrix was used, 3) changes in behavior or standard operating procedures after introduction, 4) perceived value of the matrix, and 5) program sustainability.

**Results:**

Values in the matrix were easy to calculate, but training was required to ensure correct data placement and interpretation. Displaying the matrix on a highly visible board in the maternity ward fostered a sense of accountability for health outcomes. BABIES matrix reports were compiled, reviewed, and responded to monthly by interprofessional teams, prompting collaboration across units to fill data gaps and support perinatal death reviews. Respondents reported improved staff communication and performance appraisal, community engagement, and ability to track and link clinical outcomes with actions. Midwives felt empowered to participate in the problem-solving process. Respondents were motivated to continue using BABIES, although sustainability concerns were raised due to funding and staff shortages.

**Conclusions:**

District-level health systems can use data compiled from the BABIES matrix to inform policy and guide implementation of community-centered health practices to improve perinatal heath. Future work may consider using the *Conceptual Framework on Use of the BABIES Matrix for Perinatal Health* as a model to operationalize concepts and test the impact of the tool over time.

## Background

Perinatal mortality, including newborn deaths in the first week of life and stillbirths, continues to be a significant global health threat. There are an estimated 2 million stillbirths [1] and 2.5 million newborn deaths [2] each year. Perinatal deaths are often preventable through access to high-quality antenatal, labor, and early neonatal care services [3–5], though access to such care is varied. Receipt of high-quality care practices during labor and soon after birth has been found to be positively associated with a range of maternal (married, higher age and education, employment outside the home, and urban residence), newborn (higher birthweight) and health facility/provider factors (availability of supplies, training, and supervision) [6–11].

While perinatal mortality rates have declined globally, many countries have not yet achieved the 2030 Sustainable Development Goal (SDG) target of reducing neonatal mortality to “at least as low as 12 per 1,000 live births” by 2030 [12]. The *Every Newborn Action Plan* (ENAP) provides a roadmap to accelerate progress towards ending preventable perinatal deaths by 2030, with targets addressing coverage of essential maternal and newborn health (MNH) services [13]. Uganda has not met SDG targets, where the perinatal mortality rate is estimated at 38 deaths per 1000 births, higher than the rate of 34.7 for sub-Saharan Africa [14, 15]. Well-funded, innovative solutions that help close the quality-of-care gap--such as group problem solving, community support, and health worker training--can accelerate progress towards goals in resource-constrained settings [16, 17].

Uganda is working to improve its maternal and perinatal outcomes in numerous ways, including through global collaborations. The Saving Mothers, Giving Life (SMGL) initiative was a public-private five-year partnership with the Uganda and Zambia Ministries of Health. The initiative was led by the United States Agency for International Development (USAID) and supported by the United States Centers for Disease Control and Prevention (CDC), the United States President’s Emergency Plan for AIDS Relief (PEPFAR), Merck for Mothers, and other partners [18]. It aimed to improve access to delivery care and health outcomes around the time of birth for mothers and babies in Uganda and Zambia via low cost, evidence-based interventions implemented at the district level by the Ugandan and Zambian governments [18].

**Fig. 1:**
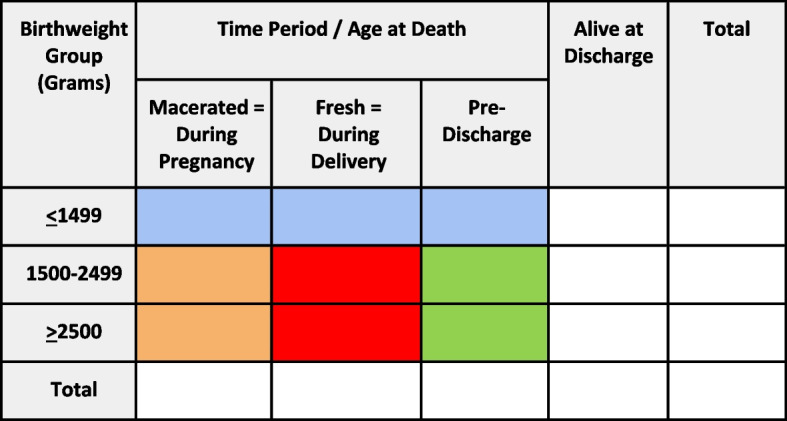
Birth weight and Age-at-death Boxes for Intervention and Evaluation System (BABIES) Matrix. NOTE: ‘Macerated’ refers to a fetal death that occurs in pregnancy before labor has started (macerated stillbirth). ‘Fresh’ refers to a fetal death that occurs during labor (fresh stillbirth). ‘Pre-discharge’ refers to a baby born alive that dies prior to discharge from the health facility (early neonatal death). These three columns sum to the total number of perinatal deaths. NOTE: The boxes are color-coded to define the timing of when interventions could be targeted for prevention. The blue-colored outcomes of very low birthweight newborns may align with interventions targeting pre-pregnancy health. The orange-colored outcomes may align with interventions targeting pregnancy. The red-colored outcomes may align with interventions during the intrapartum period. The green-colored outcomes may align with interventions in the early neonatal, pre-discharge period

One key intervention of SMGL was to improve quality of care and ensure care is evidence-based through implementation of the Birth weight and Age-at-Death Boxes for Intervention and Evaluation System (BABIES) matrix in Uganda. Rooted in a “periods of risk” analytic framework that includes both fetal and infant deaths, the matrix encourages “thinking in two dimensions” by classifying fetal and infant deaths according to both birthweight and age-at-death (Fig. 1). The color-coded outcome boxes of the matrix can be linked with prevention strategies. Higher numbers of deaths among specific weight and age-at-death outcomes can be aligned with the type and timing of interventions needed for future prevention. For example, a high number of macerated stillbirths weighing ≥1500 g suggests the need for interventions targeting maternal nutrition, prevention and management of infections during pregnancy, and quality antenatal care [19]. A high number of fresh stillbirths weighing ≥1500 g suggests the need for evidence-based interventions to improve intrapartum and delivery care (e.g., prompt recognition and management of intrapartum complications, induction of labor, vacuum extraction and C-section) [20]. The matrix was first proposed as a simple method for collecting, analyzing, interpreting, and utilizing data on perinatal deaths in developing countries [21, 22].

**Fig. 2:**
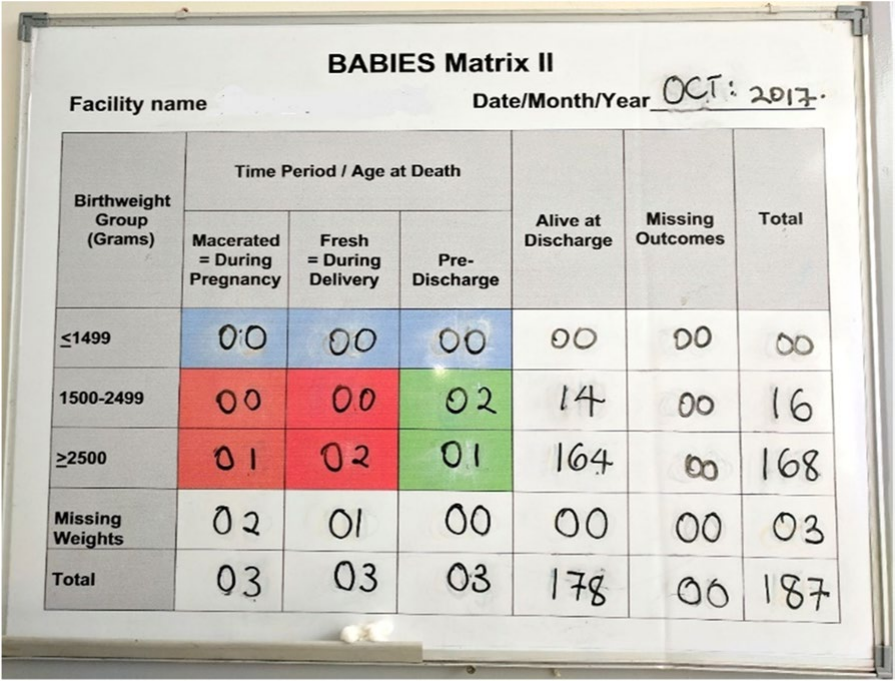
Example of BABIES Matrix displayed in a health facility maternity ward in Uganda

The matrix is designed to be adapted to local contexts and openly displayed in maternity units. Data is updated regularly to monitor monthly outcomes and can be used to complement and augment maternal and perinatal death auditing efforts. In Uganda, the matrix was reproduced on large dry eraser boards, displayed in maternity wards, completed by the in-charge for the maternity wards or other designated staff, and used for monthly monitoring (Fig. 2); paper copies of the matrix were printed for the purpose of easy tabulation. In the United States and Canada, the matrix served as the foundation of the Perinatal Periods of Risk approach (PPOR), an approach to assist community stakeholders to use local data to investigate and address fetal and infant mortality [23, 24]. In other settings, BABIES has been used to document underreporting of infant deaths [25] and to improve facility [26] and community [27] surveillance of maternal and perinatal deaths. SMGL used BABIES to leverage health facility surveillance data to guide quality improvement activities by linking maternal and perinatal health outcomes with high-quality, evidence-based health services [28].

At the end of the SMGL initiative, the Ugandan study team set out to document the experience of facility staff using the BABIES matrix. The study aim was to better understand how the BABIES matrix was used in Uganda, its perceived ease of use and value, behavior changes that resulted from its use, and whether facilities intend to continue using it at the end of the initiative. A secondary aim was to develop a conceptual framework to visually represent how use of the BABIES matrix influences maternal and perinatal health services and outcomes.

## Methods

### Study design and setting

A facility-based qualitative evaluation was conducted using in-depth interviews with individuals and small groups (up to 3 people) of health providers in 16 health facilities in the Western Region of Uganda, November 2017 [28]. Facilities included 11 health centers and 5 hospitals, including 12 public, three private not-for-profit, and one private. Participating facilities were selected by the SMGL team based on provision of Comprehensive Emergency Obstetric and Newborn Care (EmONC) services, and later expanded to include facilities providing Basic EmONC facilities. Respondents consisted of health facility staff who were actively involved with the implementation, integration, and/or use of the BABIES matrix.

The study was carried out in the SMGL-supported districts in Western Uganda, which included four contiguous, predominantly rural districts selected by the Uganda Ministry of Health to pilot the SMGL initiative framework and interventions [29]. The area is rural with low population density, mountainous topography, and limited paved roads [30, 31].

### Data collection and analysis

Interviews followed a semi-structured, in-depth interview guide developed in collaboration with the SMGL program and evaluation team. At least one interview was conducted at each facility participating in the SMGL project. Following informed consent procedures, interviews were conducted face to face by an interviewer with prior qualitative research expertise and training to ensure the quality of data. Interviews were carried out in English, audio recorded, and transcribed verbatim; the interviewer also took notes throughout the interview process. Transcripts were uploaded to a qualitative analysis software program, MAXQDAv18 for analysis. The study was reviewed by the CDC Human Research Protection Office as one aspect of the SMGL evaluation and determined to be public health practice; the Uganda MOH procedures for protecting human rights in research were followed. Ethical clearance was also given by the Makerere University School of Public Health, Higher Degrees, Research and Ethics Committee (HDREC; approval #156) on January 19, 2012 and by the Uganda National Council for Science and Technology (UNCST; approval #2927) on August 23, 2012.

Transcript data were analyzed using a directed content analysis approach [32] focused on five a priori domains: 1) perceived ease of use of the BABIES matrix, 2) how the BABIES matrix was used, 3) changes in behavior or facility standard operating procedures (SOP) after introduction of the matrix, 4) perceived value of the matrix, and 5) program sustainability. First, two reviewers read each transcript to become familiar with the content. Next, the reviewers independently coded the same initial transcript according to the five domains; they met to compare coded segments from this initial transcript and to resolve any differences. The remaining transcripts were evenly divided and coded independently by one reviewer. The interview segments were then exported into Microsoft Excel files to identify sub-themes for each domain. Reviewers met with each other to come to consensus on sub-themes and to summarize with representative quotes.

## Results

Interviews were conducted with a total of 29 health facility staff. Respondents held positions as maternity-in-charge (9), midwife (15), medical officer/physician (3), nurse (1), and data support technician (1); they had between 5 months and 20 years’ experience in health care. The interviews ranged from 25 to 73 minutes in length.

### Theme 1. Perceived ease of use

Values in the Matrix were easy to calculate, but training was required to ensure the correct placement and interpretation of data. Most respondents reported that they were initially confused by the BABIES matrix until they received proper training. Once trained, they considered it very easy to complete. The SMGL mentors were viewed as valuable for training and ongoing support.

*“...before,…people thought this was double work or added work, but as time went on, they picked up and they had a positive attitude.” - Respondent*

*“It is very easy! I learned it the very first time!" - Respondent*

*“By the time [the BABIES matrix] was introduced, they would tell us to update it on a daily basis. But it was difficult for some midwives, whereby maybe you would find someone doesn’t know what to do, then you find her not updating it, and we would always come out with missing data at the end of the month. Then the [SMGL] mentors came in and told us ‘let us try to update it at the end of the month’, and which we are doing right now…” – Respondent*

*“Ah, for the board. When the information is already collected, it is very easy to…write it on the board.” - Respondent*

The BABIES matrix was displayed on a highly visible board in the maternity ward; respondents reported that it served as a reminder of maternal and perinatal health outcomes, and as a prompt to capture and input data. Respondents described that the information displayed openly on the board made it easier to visualize the gaps; this reinforced the perception that the BABIES matrix is easy to use.

*“As we have been appreciating the matrix, to me it has really helped us a lot to identify very many gaps.” - Respondent*

### Theme 2. How the BABIES matrix was used in the facility

Respondents reported that midwives played a significant role in implementation of the BABIES matrix, including scheduling monthly meetings and collecting, computing, and reporting data. They explained that midwives were often assisted by other health workers, such as fellow midwives, students, nursing assistants, and obstetricians; some facilities had data specialists to manage the data.

In nearly all of the participating facilities, reports for the BABIES matrix were compiled and reviewed around the end of each month. This process prompted engagement between the maternity, nursery, and neonatal intensive care units to fill data gaps in newborn weight and outcomes and helped to prompt initiation and completion of death reviews, usually in separate meetings. Respondents indicated that the time required to fill out the monthly reports for the BABIES matrix ranged from very quick when completed all at one time (10–15 minutes) to up to 1 week when done intermittently. The dedicated time spent often depended on resources such as computer software and time. All who were involved with the BABIES matrix, including administrators (e.g., Medical Director and Senior Nurse Officer), attended the monthly meetings to discuss outcomes and trends.

*“The Senior Nurse Officer [Maternity-in-Charge reviews] on a monthly basis, I am the one who does what, check through the documentation, how many babies were born according to kilogram body weight. And I go to NICU [neonatal intensive care unit] to some more of those pre-discharge, so we can what, include them on our matrix.” - Respondent*

*“[W]e usually review it in the monthly meetings. It is an integrated meeting whereby we have maternity staff and NICU staff. I (the Nurse officer/Midwife/Maternity-in-charge) am the chairperson, so we make an agenda, then we reach the point where we review the data. Sometimes we find we have so many macerated [stillborn], then we talk about that one. Sometimes there are [neonatal deaths]. And we find out how many have not been audited. We also talk about if we have audited them, then we check the days, and we see how we audit those deaths.” - Respondent*

### Theme 3. Changes in behavior or facility SOPs

A team-based approach was required and reinforced by the BABIES matrix; respondents explained that the tool required the effort of many individuals to complete and provide an appropriate response. Respondents reported that this collaborative strategy changed the way staff interacted and communicated with each other. It allowed open discussion, accountability for, and resolution of issues evidenced by the data. Respondents described that when problems were addressed, particularly newborn and maternal deaths, the discussions were supportive and focused on problem solving versus blaming, shaming, and punitive actions.

*“[The Ministry of Health] congratulate[s] us, then they say we have improved from what we were doing to a certain level. But if it is not that, they also tell us that the other time [before BABIES matrix] we discussed this and this, we said you can improve by doing this and this, but it was not done [previously].” - Respondent*

One facility was intentional about engaging with their community because of what was learned at monthly meetings. Respondents from this facility described that the BABIES matrix provided tangible evidence of the outcomes that could be reported back to the community. As specific issues were identified by the team through use of the tool, community-based interventions were developed with community partners.

*“In antenatal we have educated the mothers to do antenatal early, to start as early as they know they are pregnant and informed the VHTs [Village Health Teams] to refer us mothers who are pregnant in the community as early as possible, so they start antenatal early and we do what, we do the tests like syphilis test, HCT [HIV counseling and testing]. If they are syphilis [positive], we treat early, as early as possible, so we avoid those macerated stillbirths.” - Respondent*

Sometimes the BABIES matrix did not entirely replace the existing procedures and processes but was integrated into existing practices.

*“In our meetings we always have a performance monitoring tool, whereby we make sure we give people some duties which we have to monitor at the end of the month…and we sometimes use quality improvement tools. We make like a journal, so this time we are going to improve on this, so at the end of the month, we come back to see have we achieved anything, or we have relapsed.” - Respondent*

### Theme 4. Perceived value of the matrix

Respondents reported initial apprehension about implementing the BABIES matrix due to a perceived increase in workload quickly progressed to acceptance. They reported improved outcomes including more transparent communication and performance appraisal among staff, increased engagement with the community for education, and the ability to track clinical outcomes and link them to specific clinical actions. Staff, especially midwives, shared their experiences of feeling empowered to actively participate in problem-solving and speak up, even in the presence of higher-level staff. These experiences contributed to increased accountability among team members.

*“People appreciated that it was pointing to something. It was just not numbers on the wall and in boxes. Yeah, we were interpreting out what exactly is happening, and the other part is it was involving people, it was giving people a chance to suggest, because during the review meeting we tried to allow people to criticize even the medical officer, if he has been doing something wrong, we try to open it up and say, okay you’re free still, me I [the Medical Officer] will make sure that they cool the guys down, but please tell me if they think a medical – a particular medical officer was not doing what they wanted, or they called and he took an extra minute to come, or whatever, so these kind of things where it somehow told people it’s just not the work, but it is helping us to be better, so somehow a few people felt, okay we could try this.” - Respondent*

*“They are responsive, and the fact is that these days they encouraged us to talk about performance. So every time we are in the morning, we are in those meetings, and I share with them about this performance, everybody is always glad to know how they are performing.” - Respondent*

While respondents reported appreciating the BABIES matrix, they offered some suggestions to strengthen the tool. Recommendations included adding a comment section for supplemental information, implementing periodic training for new staff and refresher sessions, and inclusion of other data elements relevant to perinatal and maternal outcomes.

*“It’s perfect! Me, I (a midwife) see it is capturing everything that we want.” - Respondent*

*“Maybe we would also include the exposed infants. The number of the [HIV] exposed infants…babies born to HIV positive mothers. Because as we talk, we’ve spent some good time without the nevirapine syrup for these babies, so maybe if we, those numbers are shown there, people can feel it, they can know, ‘Hey this is a big number, I think they should get some care’.” - Respondent*

*“Maybe making it more useful we need some refresher trainings... Because some people forget, you find that, ‘Oh, here she forgot.’ Or here I forgot, so it can work better with refresher trainings. Because you may find that somebody came from a lower health center, and now she’s upgraded to the higher health center, so [she] doesn't know.” - Respondent*

### Theme 5. Sustainability of the program

Many health workers indicated that the BABIES matrix use had become a habit in their facility. Training sessions increased familiarity and confidence in continued use of the BABIES matrix. Staff reported motivation to continue compiling data points and completing the matrix by their recognition of improved perinatal and maternal health outcomes perceived to result from using the matrix.

*“…It will be our mandate to still continue this because we know the importance of it.” - Respondent*

*“For us we’ve not even felt that it is SMGL which brought it, we feel it is part of us.” - Respondent*

For some facilities, the government-employed health workers affiliated with SMGL will continue as a part of the team even after SMGL ends. They will continue to mentor and serve as a liaison between the facility and the government.

*“…Now we are working side by side with the government employees, those that we are working with now…will remain after SMGL has gone…and she will continue to mentor whoever new additions of midwives, in all, not only in matrix, BABIES matrix, but in all obstetric care.” - Respondent*

While respondents spoke positively about their intentions to continue using the BABIES matrix, many cited staffing shortages and lack of continued support as potential barriers. Most respondents indicated that they will not have continued mentorship and supervision after SMGL ends, which would negatively affect their use of the BABIES matrix, regardless of intentions.

*“Like their position is financed by SMGL, and they said that that was a concern, because if there’s fewer staff then it might be more difficult to keep the matrix going.” - Respondent*

*“Yeah, it may continue. It may continue because people are trained, and it may not continue because they don’t have supervisors.” – Respondent*

### Conceptual framework for the BABIES matrix implementation

**Fig. 3:**
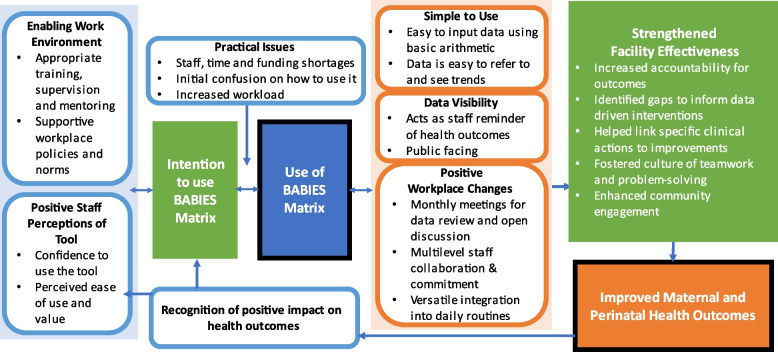
Conceptual Framework on Use of the BABIES Matrix for Perinatal Health

A conceptual framework, informed by the qualitative results, was developed to display the relationships among key domains (Fig. 3). These relationships are described below:

- An enabling work environment (training, supervision and mentoring, supportive policies and norms) and positive staff perceptions of the tool (confidence to use the BABIES matrix, perceived ease of use and value) strengthen intention to use the BABIES matrix. In response, increased intention to use the BABIES matrix may improve the enabling work environment and positive staff perceptions of the tool.
- Intention to use the BABIES matrix leads to initiation and continued use of the BABIES matrix. In response, use of the BABIES matrix may increase intention to use the BABIES matrix. The influence of intention to use the BABIES matrix on actual use of the BABIES matrix is moderated by practical issues (staff, time and funding shortages, initial confusion on how to use it, increased workload).
- Use of the BABIES matrix leads to staff perception that it is simple to use, increases data visibility (staff reminder of health outcomes and visible to community members), and promotes positive workplace changes (monthly meetings for data review and open discussion, multilevel collaboration and commitment, versatile integration into daily routines). In response, staff perception that it is simple to use, increased data visibility, and promotion of positive workplace changes may increase use of the BABIES matrix.
- Through perceptions that it is simple to use, increased data visibility, and positive workplace changes, use of the BABIES matrix leads to strengthened facility effectiveness (increased accountability for outcomes, identified gaps to inform data driven interventions, linked clinical actions to improvements, fostered culture of teamwork and problem solving, enhanced community engagement).
  - ◦ Strengthened facility effectiveness may contribute to improved maternal and perinatal health outcomes.
- Recognition of improved maternal and perinatal health outcomes reinforces positive staff perceptions of the tool and intention to use the BABIES matrix.

## Discussion

Implementation of easy-to-use, community-based surveillance systems in Uganda, and in other resource-constrained areas, is a growing need. The aim of perinatal health surveillance systems is to prompt quality improvements in maternal and perinatal health services over time. Recent initiatives highlight the challenges faced and the benefits of using evidence for quality improvement [19, 20, 33, 34]. The BABIES Matrix surveillance methodology presents an opportunity for district and facility-level health systems to consistently collect robust and actionable surveillance data. Our qualitative findings suggest that, with a supportive work environment and proper training and mentorship, the BABIES Matrix is perceived as a valuable and simple tool to help identify and fill gaps in quality health services. The visibility of health outcomes data, combined with structured workplace changes, fosters accountability and teamwork, prompting improved health facility effectiveness.

High levels of support for the BABIES matrix were found in health facilities participating in the study. For some staff, there were sustainability concerns due to potential limited staffing and financial resources after the SMGL initiative ends. Health systems in other resource-constrained settings cite limited staffing and financial resources as major barriers to quality improvement programs [32]. Fortunately, evidence of ownership and integration of the BABIES matrix into staff routines and positive shifts in workplace culture suggests there is momentum for this intervention beyond the initial investments. As staffing levels affect use of the BABIES matrix tool, strategies to effectively train, maintain confidence and skill, and engage staff in its use are important. Consistent integration of the BABIES matrix activities into expected clinical and supervisory duties, paired with a funding commitment, could promote longevity of the system. Further research is needed to document the mid- and long-term use of the BABIES matrix following the end of the SMGL project and to better understand the barriers and enablers for its sustainability.

The versatility of the BABIES matrix was evidenced by its use among diverse health facility staff (e.g., midwives, maternity in charge, administration) and frequency of its use. Several facilities cited that the BABIES matrix was regularly discussed in conjunction with perinatal death reviews or other pre-existing meeting structures. The matrix required greater detail than previously completed monthly reports, providing an opportunity for consistent information to be tracked over time and across facilities. Collectively, responses suggest that the matrix contributed to a standard practice of using data and addressing missing data. Review of the BABIES matrix was used as a learning opportunity, and several facilities cited the importance of a team-based approach with designated staff responsible for completion. These facilities used a non-punitive, non-judgmental approach in discussing health outcomes and developed team-oriented solutions. These strategies align with documented approaches for health services quality improvement [35].

The BABIES matrix was implemented with the intention that it would be integrated into facility processes and sustained beyond the SMGL initiative. Discussion of the tool at meetings with diverse levels of staff showed evidence of increased buy-in of the process over time and perceived value of the matrix. Additional evidence for the usefulness of the BABIES matrix comes from its recommendation for scale-up in the *National Maternal and Perinatal Death Surveillance and Response (MPDSR) Guidelines* [36]*.*

Several facilities emphasized the visual impact that the BABIES matrix had as a daily reminder of quality improvement progress. This is especially relevant for facilities where death audit discussions are limited to leadership or only those directly involved in the case. The value of visual aids as effective tools to improve decision making has also been highlighted in other work [37]. Continuing the matrix team-training approach and integrating it into orientation for new staff may help cultivate positive perceptions of the tool, thereby fostering inclusivity and motivation, and creating champions for the work. Such approaches have been linked with improved client health, staff, and organizational outcomes [38].

Completing the BABIES matrix helped to bridge gaps in maternal and perinatal data. Maternity ward staff described increased communication with NICU units to ensure accuracy of newborn outcome data. The implementation of the matrix encouraged active use of data for action, particularly among midwives, and promoted a non-punitive environment of teamwork for collective problem-solving. There was also evidence of local and district level administrators and health workers reaching out to communities through village health teams based on information collected in the matrix. This linkage, prompted by use of the BABIES matrix, speaks to the value of chronologically and geographically contiguous surveillance system data. One facility described a “full circle” approach where results from the BABIES matrix were shared with the community. In turn, the community, via communication with community health workers, provided context for the surveillance data, and in some cases, helped to explain the potential causes of adverse outcomes. This type of community engagement sets the foundation for more in-depth consultation with communities where members can help co-design and test interventions [39].

A perinatal surveillance system can support the development and implementation of health interventions by tracking outcomes over time and providing actionable data. The PPOR approach by CityMatCH (a National Organization of Urban Maternal and Child Health Leaders) is one such system adapted from the BABIES matrix and implemented across cities in the USA with high infant mortality rates [23]. The continuity of data across facilities and districts/states provides an opportunity to compile and compare information for mutual learning. While the specific contribution of the BABIES matrix intervention cannot be quantified, the SMGL initiative was found to be cost-effective and contributed to declines in institutional perinatal deaths [29, 40]. Implementation of the BABIES matrix could be expanded to other resource-constrained settings to improve data collection and use of data for action at the local level. Best practices from this evaluation may be useful to facilities and jurisdictions implementing the BABIES matrix surveillance system (Table 1). Future work may consider using the *Conceptual Framework on Use of the BABIES Matrix for Perinatal Health* as a model to operationalize the concepts and test the impact of the tool over time.

**Table 1:** Best Practices for Implementation of the BABIES Matrix

Best Practice	Description
Supportive workplace policies with multilevel collaboration	Regular discussions of the BABIES Matrix data that include all levels of staff can foster an environment of learning and help to integrate the use of the matrix with other quality improvement processes (e.g., perinatal death reviews).
Enabling work environment with appropriate training	Recurrent training/refreshers and integration into new staff orientation supports team member inclusivity, availability of trained staff, team spirit, and motivation to track outcomes.
Identify and support champions	Appoint a designated person responsible to collect data and complete the matrix. Ensure this person can delegate the task or receive assistance from helpers (e.g., students, records staff). Integrate BABIES matrix duties and processes into standard operating procedures.
Collect and increase visibility of surveillance data	Visible changes in adverse outcomes on the BABIES matrix prompts facilities to work with their communities to identify potential causes and to foster a sense of agency and accountability over health challenges.

### Limitations

Interpretation of the study findings are limited as the study took place in one region of Uganda with a relatively small number of respondents. In addition, the study team-defined topics and interpretations may differ from those of the respondents (e.g., interpretation of *perceived value* as expressed feelings towards the tool versus feelings towards the resulting processes). Lastly, social desirability bias may have resulted in respondents sharing what they perceived to be more acceptable answers.

## Conclusions

The BABIES matrix is a simple tool that was created to provide a standardized overview and quick assessment of perinatal health outcomes so that quality improvement strategies can be implemented. Experiences from select facilities support the potential of the tool to increase the capacity of local health systems to coordinate, plan, and evaluate health interventions, and to interpret and use data for action. Midwives felt empowered to actively participate in the problem-solving process. In addition, health facility teams experienced increased accountability for perinatal health outcomes. With the support of government officials and public health leadership, district-level health care systems can harness these data to inform policy and guide the implementation of community-centered health practice to prevent perinatal deaths.

## Data Availability

Qualitative data are available from the CDC Division of Reproductive Health upon request. Requests should be directed to Florina Serbanescu.

## Abbreviations

BABIES: Birth weight and Age-at-death Boxes for Intervention and Evaluation System
BWPMR: Birth weight proportionate mortality rates
BWSMR: Birth weight specific mortality rates
CDC: United States Centers for Disease Control and Prevention
EmONC: Emergency Obstetric and Neonatal Care
ENAP: Every Newborn Action Plan
HCT: HIV counseling and testing
HDREC: Higher Degrees, Research and Ethics Committee
MPDSR: Maternal and perinatal death surveillance and response
MNH: Maternal and newborn health
MOH: Ministry of health
NICU: Neonatal intensive care unit
PEPFAR: United States President’s Emergency Plan for AIDS Relief
PPOR: Perinatal Periods of Risk
QI: Quality improvement
SDG: Sustainable Development Goal
SMGL: Saving Mothers, Giving Life
SOP: Standard operating procedure
UNCST: Uganda National Council for Science and Technology
USAID: United States Agency for International Development
VHT: Village Health Team
